# Influence of CYP2C8 Polymorphism on the Exposure to Chloroquine in Patients with Malaria by *Plasmodium vivax*—A Preliminary Study

**DOI:** 10.3390/ijerph22030336

**Published:** 2025-02-25

**Authors:** Luann Wendel Pereira de Sena, Hellen Thais Fuzii, Fabiola Elizabeth Villanova, Amanda Gabryelle Nunes Cardoso Mello, Maria Pantoja Moreira de Sena, Michelle Valéria Dias Ferreira, José Luiz Fernandes Vieira

**Affiliations:** 1 College of Collective Health, Federal University of the South and Southeast of Para, Cidade Universitaria, Avenida dos Ipês s/n, Maraba 68500000, Para, Brazil; 2 Tropical Medicine Center, Para Federal University, Generalissimo Deodoro Street 92, Belem 66074740, Para, Brazil; 3 Pharmacy Faculty, Para Federal University, Campus Universitario do Guama, Augusto Correa Street 01, Belem 66074740, Para, Brazil; 4 Postgraduate Program in Tropical Diseases, Para Federal University, Generalissimo Deodoro Street 92, Belem 66074740, Para, Brazil

**Keywords:** malaria, polymorphism, chloroquine, *Plasmodium vivax*

## Abstract

Aim: To assess the impact of the CYP2C82 polymorphism on chloroquine and desethylchloroquine concentrations in patients with malaria caused by *P. vivax*. Methods: A prospective study was conducted on patients with malaria in an endemic area of the Amazon basin. Liquid chromatography was employed to measure the levels of chloroquine and desethylchloroquine, while molecular methods estimated the frequency of the CYP2C82 variant. Results: This study revealed that plasma levels of chloroquine were higher in patients with the CYP2C82 polymorphism compared to those without this variant. The difference in plasma levels ranged from 5% to 26.5%. Conversely, patients with the CYP2C82 polymorphism exhibited lower levels of desethylchloroquine. Conclusion: The findings of this study confirm the impairment of chloroquine metabolism by the CYP2C82 variant. However, it is noteworthy that in the dose regimen used for malaria treatment, these changes did not lead to toxic concentrations of the drug.

## 1. Introduction

Chloroquine is a component of the first-line treatment for malaria caused by *P. vivax*. The recommended total dose is 25 mg/kg, taken over three days [1]. This dosing regimen is considered safe, with most patients experiencing only mild adverse effects, such as gastrointestinal discomfort (nausea, vomiting, anorexia, abdominal pain), dizziness, pruritus, and headache [2]. However, the drug has a narrow safety margin, and high doses can lead to serious adverse effects, including potentially fatal cardiac arrhythmia [3,4]. Exposure to high doses usually occurs due to intentional or accidental misuse [3]. Recently, some studies have raised concerns about the use of high doses of chloroquine in clinical trials for COVID-19, which have resulted in episodes of serious cardiac abnormalities [3,4].

Chloroquine undergoes N-desethylation to form desethylchloroquine primarily through CYP-450 enzymes such as CYP2C8 and CYP3A4, and to a lesser extent by CYP2D6 [5]. These enzymes are subject to genetic polymorphisms that can abolish, reduce, or increase their activities [6]. A matter of interest is whether the polymorphisms associated with low enzymatic activity can lead to toxic levels of the drug. For instance, CYP2C8 appears to be the most important pathway of chloroquine metabolism, and its most prevalent polymorphisms are CYP2C8*2, CYP2C8*3, and CYP2C8*4, which have been associated with reduced metabolism of several drugs [6].

Currently, there are limited data available on the plasma levels of chloroquine in patients with the CYP2C8*2 variant, which is relevant to assess whether these patients are at an increased risk of experiencing serious adverse effects. Therefore, this study aims to estimate the changes in the plasma levels of chloroquine in patients with *P. vivax* malaria who possess the CYP2C8*2 variant.

## 2. Methods

A prospective study was conducted in the municipality of Anajas, Brazil, from January 2018 to July 2019. This study included male patients aged 18 years or older, who tested positive for *P. vivax*. Exclusion criteria comprised patients with signs or symptoms of severe malaria, those with hypersensitivity to chloroquine, those who had received anti-malarial treatment within three months before the study’s commencement, patients with CYP2C8*3 and CYP3A4*1 variants, and those who declined to provide consent or did not sign the consent form.

Each patient was treated with chloroquine diphosphate at doses of 10 mg base/kg on the first day followed by 7.5 mg/kg on the second and third days, combined with primaquine diphosphate at a dose of 0.50 kg base/kg for 7 days [1]. The administration of these drugs was strictly supervised by the research team. To monitor potential adverse effects, particularly vomiting, patients were observed up to 2 h after drug intake. Follow-up evaluations were scheduled at 24, 72, and 672 h after clinical assessment and collection of blood samples.

Venous blood samples (5 mL) were taken for the measure of analytes. A portion of whole blood was separated for genotyping. The quantification of chloroquine and desethylchloroquine was performed in plasma using a reversed-phase High-Performance Liquid Chromatography (HPLC) system with fluorescence detection (Flexar, Perkin Elmer™, Shelton, MA, USA), following the procedure proposed by Pham et al. (2016) [7,8]. Genomic DNA was isolated from peripheral blood using the DNeasy^®^ Blood & Tissue kit (QIAGEN™, Düsseldorf, Germany) and the polymorphism was genotyped in the CYP2C8 gene (GenBank accession number rs11572103, T > A) by real-time PCR using specific hydrolysis probes for the SNP assay (Applied Biosystems™, Foster City, CA, USA). The TaqMan assay follows the recommendations of the manufacturer. Positive and negative controls were included in each analysis (Applied Biosystems, AB™).

Parasite identification and quantification were performed using the Giemsa-stained thick blood smear technique (pH = 7.2). The slides were examined on the same day the smears were prepared. A total of 100 microscopic fields (equivalent to 0.2 mm^3^ of blood) were scanned.

Parasitemia was estimated by counting asexual parasites per 200 leukocytes. If fewer than 10 parasites were detected, counting continued up to 500 leukocytes. Conversely, if the microscopic count exceeded 500 parasites before reaching 200 leukocytes, counting was halted after the final field was read.

The data are presented as median and range or as a frequency of occurrence. To compare the concentrations of analytes between the genotypes on each day of blood sampling, the Mann–Whitney U test was utilized. Allele and genotype frequencies were estimated through gene counting. Deviation from the Hardy–Weinberg equilibrium was assessed using the Chi-square test with Bonferroni correction, employing Genepop software (Genepop C++ version 4.7.0). The accepted significance level was set at 5%.

This study was revised and approved by the Ethical Committee of the Health Science Institute of the Federal University of Pará, number 2.819.240.

## 3. Results

A total of 210 patients were enrolled in this study. The mean age was 32 years (range, 27–45 years). Among them, 13 patients (6.19%) were found to have the CYP2C8*2 variant. The genotype distribution did not significantly deviate from the Hardy–Weinberg equilibrium (X^2^ = 0.2245; *p* = 0.6356). To prevent bias related to sample size in the comparisons of drug levels between genotypes, a sub-sampling of patients (n = 13) was randomly selected from those who did not possess the investigated variant, including CYP2C8*3 and CYP3A4*1. The geometric mean of parasitemia at admission was 1132 (6.1) parasites/µL in patients with the CYP2C8*2 allele and 1380 (5.4) parasites/µL in those without the polymorphic allele (*p* > 0.05). During treatment, parasites were cleared from the blood in both genotypes within a median time of 96 h. Furthermore, there was no reappearance of parasites in peripheral blood during the follow-up period in either genotype.

Table 1 presents the median plasma concentrations of chloroquine, desethylchloroquine, and the ratio of the metabolite-to-parent drug for each genotype on each day of blood sampling. The baseline samples did not show any measurable concentrations of the drug or its metabolite.

## 4. Discussion

The frequency of CY2C82 was found to be 6.19%, which is consistent with a large population survey conducted in the north region of Brazil, where frequencies of 6.4% and 7.5% were reported in white and black individuals, respectively [9]. To minimize potential variations in the expression of CYP enzymes related to sex, this study specifically included only male patients [6].

The highest concentration of chloroquine occurred at 72 h in both genotypes, which is consistent with the drug’s characteristics of extensive distribution, accumulation in melanin-rich tissues, and long half-life [5]. The levels of desethylchloroquine were significantly lower at 24 and 72 h. Patients with the CYP2C8*2 variant exhibited impaired chloroquine metabolism, as evidenced by elevated chloroquine levels throughout the blood sampling period, accompanied by reduced levels of desethylchloroquine. Furthermore, the metabolite-to-parent drug ratio was significantly lower in patients with the CYP2C8*2 variant at 24 and 72 h.

Based on previous studies, the observed 26.5% increase in plasma drug levels in patients with the CYP2C8*2 variant is unlikely to cause serious adverse effects. Considering a whole blood-to-plasma concentration ratio of 4:1, the drug levels do not reach values associated with serious cardiovascular abnormalities. The median whole blood concentration of 957 ng/mL, which is commonly found in patients undergoing malaria treatment, does not cause clinically significant QRS prolongation. There may be a slight prolongation of about 6.7 msec (ranging from 5.5 to 7.8 msec), but this is not considered clinically significant. Only a QRS prolongation greater than 150 msec triggers a significant delay in intraventricular conduction, leading to important cardiac arrhythmias [3,4].

The increase in chloroquine levels associated with the CYP2C8*2 variant can potentially impact its tolerability, similar to what has been observed with amodiaquine in children from Zanzibar, where the CYP2C82 variant led to higher rates of dizziness and insomnia [10]. Furthermore, the adverse effects may contribute to non-compliance with treatment, as observed in the Brazilian Amazon basin, where they were responsible for treatment interruption in a significant portion of patients who eventually abandoned therapy [2].

The major limitation was the small number of patients with the CYP2C8*2 variant, primarily due to the low frequency of the variant in the study population. Nevertheless, the data provided support that the CYP2C8*2 variant studied does not lead to toxic plasma levels of chloroquine associated with significant cardiac abnormalities when used in the doses typically prescribed for the treatment of malaria.

## Figures and Tables

**Table 1:** Concentrations of chloroquine and desethylchloroquine, expressed as median and range, in several days of blood sampling.

Genotype	24 h	72 h	672 h
No polymorphic allele			
Chloroquine ng/mL (n = 13)	154 (77–293)	486 (221–801)	45 (37–64)
Desethylchloroquine ng/mL (n = 13)	69 (37–135)	232 (110–392)	32 (22–45)
Ratio metabolite: parent drug	0.42 (0.39–0.46)	0.40 (0.38–0.49)	0.56 (0.4–0.71)
Polymorphism CYP2C8*2			
Chloroquine ng/mL (n = 13)	161 (150–325)	615 (550–832) *	65 (36–82)
Desethylchloroquine ng/mL (n = 13)	56 (29–102) *	205 (147–321) *	30 (29–64)
Ratio metabolite: parent drug	0.35 (0.2–0.4) *	0.33 (0.27–0.39) *	0.56 (0.42–0.76)

* *p* < 0.05.

## Data Availability

The data presented in this study are available on request from the corresponding author. The data are not publicly available due to privacy and ethical restrictions.
